# Stance Detection Based on User Feature Fusion

**DOI:** 10.1155/2022/5738404

**Published:** 2022-03-30

**Authors:** Weidong Huang, Yuan Wang, Jinyuan Yang, Yijun Xu

**Affiliations:** ^1^College of Management, Nanjing University of Posts and Telecommunications, Nanjing 210000, China; ^2^College of Foreign Languages, Nanjing University of Posts and Telecommunications, Nanjing 210000, China

## Abstract

Rapid development of the Internet has contributed to the widespread adoption of social network platforms. Network media plays an important role in the process of public opinion dissemination and bears significant social responsibility. Public opinion mining is of great significance for online media to improve the quality of content provision and enhance media credibility. How to make full use of user-generated content is the key to improving the accuracy of position detection tasks. In this paper, we proposed a stance detection model based on user feature fusion by using comments of netizens in false news events on Weibo as research content. The method of feature fusion is adopted to integrate vectors including user sentiment, cognitive features, and text feature at the feature layer for model training and position prediction. The model is evaluated on a dataset of related microblog comments in false news. The result shows that our proposed method has a certain improvement in the effect of stance detection.

## 1. Introduction

With the rapid development of the Internet, news dissemination has shifted from newspapers, television, and other traditional modes to mobile internet positions. Social network platforms, such as Weibo and Twitter have permeated all aspects of life. Weibo has attracted many netizens because of its free and convenient method of information interaction. Mainstream media and we-media users play an important role as content providers in the social network platform. They publish content and disseminate information through social network platforms, which can turn social events into hot spots of public opinion and gather massive public opinions in a short time, thereby influencing the process of public opinion.

Netizens have a high degree of participation in network public opinion events, and the opinions and comments they release have significant sentiment tendencies, which reflect an attitude toward the content publisher and their experience of content reception, especially those of dissatisfaction such as query and opposition. Those sentimental comments are very likely to affect the credibility of the social media, destroy the media image, and even hinder the healthy development of the Internet. Therefore, what type of information the network media should provide, what type of image it should establish, and how to play a positive role in the dissemination of public opinion are significant issues to investigate. By collecting comments from relevant media microblogs after a false news release as the research content, the authors combine vectors including user sentiment features, user cognition features, and comment text features. In this paper, the authors introduce a stance detection model that fuses feature vectors for prediction. The purpose is to monitor the acts of internet users along with their attitudes toward false news released by content providers and to provide a basis to improve the quality of internet content and media value.

## 2. Related Work

### 2.1. Research on Stance Detection

Stance detection and sentiment analysis are two important research directions in the field of public opinion mining. Previous research studies on stance detection mainly focused on the content of English online forums and political debates, such as stance detection on political figures, policies and regulations, public events, and products, and was later applied to the field of public opinion analysis [1]. According to the characteristics of the public opinion corpus, stance detection mainly involves three aspects:

- Text-based semantic mining: convolutional neural networks (CNNs) have shown superior performance in many text classification tasks, such as sentiment classification and false news detection, and it is widely used in stance detection. Poddar et al. [2] encoded tweets using CNN with attention mechanisms to force the neural network to focus on parts of the tweet that are important for determining its instance. Considering the importance of event background for stance detection, Zeng [3] proposed a CNN-gated recurrent unit (CNN-GRU) stance detection model, which combines grammatical and event-related features of microblog texts. With the development of sentiment analysis in natural language processing research, some scholars have noticed that the user's emotional information is meaningful for stance detection. Considering the correlation between stance and sentiment, Sun et al. [4] proposed a novel neural network model to jointly learn sentiment features and stance features. However, part of the emotional information will be lost after the learning of the neural networks, and the sentiment feature representation is incomplete. Therefore, how to make full use of sentiment features to effectively promote the effect of stance classification remains to be further studied.
- Stance detection considering corpus structure: many scholars have noticed the influence of corpus structure as the study of stance detection progressed. Li [5] used LSTM to realize the annotation of the conversation sequence and proposed a stance detection model, which considered the tree structure to fully utilize the original rumor information and the dialog structure of the corpus. Poddar et al. [2] used recurrent neural networks (RNNs) with tweet-level attention mechanisms to capture dialog sequence features for tweet stance detection.
- Stance detection based on feature engineering: because the focus of the preceding two aspects is still limited to text, some scholars attempt to introduce the characteristics of other dimensions in stance detection to improve the classification effect [6]. Xuan [7] selected 18 features for stance detection from three dimensions, such as text, user, and communication. Wang et al. [8] introduced systemic functional linguistics (SFL) theory in stance detection research, designed a feature combination of conceptual metafunction, textual metafunction, and interpersonal metafunction, and screened the optimal features through statistical and visual analysis to extract features from user reviews comprehensively and multidimensionally. In addition to features such as sentiment, content, and part of speech, Ayyub et al. [9] used in-depth features (such as GloVe and Word2Vec) for classifier training. In order to obtain the optimal combination of feature templates and classifiers, Xu et al. [10] used two feature selection strategies (top *k*-based selection and leave-out *k*-based selection) to generate the optimal feature set. Although the feature template is extended, the abovementioned method relies primarily on the statistics of original data, resulting in numerous calculations and reducing the real-time performance of the stance detection system. Therefore, some scholars have considered multidimensional information fusion methods to improve the performance of stance detection.

### 2.2. Feature Fusion

In 1973, the US Department of Defense funded sonar signal processing systems, which pioneered information fusion, also known as data fusion. It can be divided into three levels: data fusion, feature fusion, and decision fusion. With the development of deep learning technology, the feature fusion has more significant advantages. The information fusion of the feature layer can be divided into early fusion and late fusion based on the sequence of fusion and prediction.

- Early Fusion: the fusion is performed at the feature-level first, and then the fused features are input into a model for training. Concatenation (concat) and addition (add) are the two most common fusion methods [11]. The concat method directly splices two feature vectors, and the add method combines two feature vectors into a complex vector. Chen et al. [12] proposed a text detection method based on multigranularity feature fusion by combining multigranularity and cognitive knowledge. The model not only fully learns the information about different granularity features in the image but also pays more attention to the target feature information and suppresses useless information, which improves the robustness and accuracy of the model by fusing different granularity features of the general feature extraction network and adding the residual channel attention mechanism. To avoid the addition of irrelevant information and data redundancy, a threshold-based parallel fusion method is proposed, which fuses multidimensional features into a vector and then selects the most prominent features for final classification [13].
- Late Fusion: the performance is improved by fusing the results of different layers. There are two main representatives of this method. One is feature nonfusion. The multiscale feature is used to predict separately, and then the prediction results are fused, such as single-shot multibox detector (SSD) [14] and multiscale CNN (MS-CNN) [15]. The other adopts the idea of a feature pyramid network (FPN) [16], where the prediction is performed after feature fusion. Based on the deep forest, a fusion-enhanced cascade model (FECM) is proposed, which fuses the surface features from the multigranularity module and combines the cascaded gradient descent tree (GBDT) and random forest models to fuse the features [17]. Zhang et al. [18] proposed a train spatiotemporal graph convolutional network (TSTGCN) that includes two parts, spatiotemporal attention mechanism and spatiotemporal convolution. This method is used to capture spatiotemporal characteristics in three levels; therefore, the final result is predicted by the weighted fusion of the three components. Gunes and Piccardi [19] used two different strategies. One is to fuse “Face” features and “Body” features into a feature and train the classifier for classification, which is “feature fusion.” The other is to select the appropriate classifiers for “Face” and “Body.” The classification results of the two are fused to obtain the final result, which is “decision fusion.” In the author's experiment, “feature fusion” performs better.

Feature fusion reduces the workload of original data processing and improves the system processing speed and real-time performance.

Stance detection must mine users' attitudes, which are more complex than sentiment analysis, and are influenced by their cognition. Therefore, the authors propose a method based on the fusion of comments text and users' sentiment, as well as cognition features to conduct stance detection research on Weibo user comments during the dissemination of fake news.

## 3. Problem Formulation

In public opinion network events, media users, as content providers with significant influence, play an important role in the process of public opinion dissemination. Simultaneously, the opinions and comments released by netizens not only reflect their attitudes toward the incident but also reflect their attitudes and stance toward media and we-media users. Particularly, dissatisfaction, such as queries and oppositions, affects media images. The authors conduct stance detection research on the comments under relevant microblogs to further explore the content perception experience brought by media users to netizens.

### 3.1. Stance Classification Settings

The content of Weibo comments reflects the attitude toward the authenticity of the source rumor microblogs. Generally, stance classification tasks classify stances into four types, such as support, comment, query, and deny, in which comment is a neutral stance without bias.

- Support: the stance that has no doubt or opposition to the authenticity of the source microblog and consistent with the theoretical proposition of the source microblog is the support stance.
- Comment: the stance that has no doubt or opposition to the authenticity of the source microblog is the comment stance.
- Query: the stance that expresses doubts, inquiries, or verification of the relevant information about the authenticity, scientificity, and rationality of the source microblog shall be regarded as query stance.
- Deny: the stance that refutes the authenticity, scientificity, and rationality of the source microblog shall be regarded as deny stance. Oppositions not involving true or false judgments, such as opinions on whether the content of the source microblog is ethical or not, are not classified as deny stance.

### 3.2. Correlation Analysis of User Features and Stance

#### 3.2.1. Sentiment Stance

**Figure 1:**
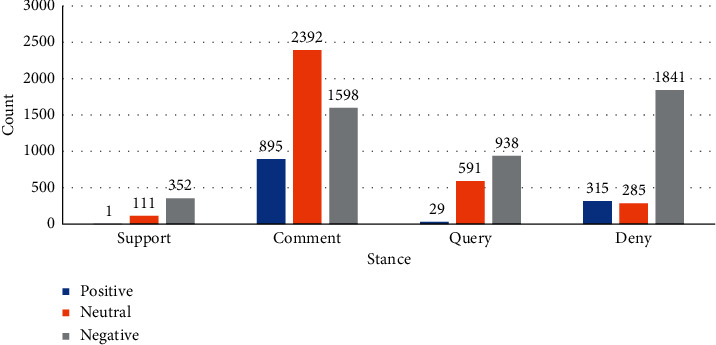
The distribution of sentiment features in different stances.

Stance detection must mine user attitudes, which are the expression of sentiments but more complex than sentiment analysis. Some online media deliberately enlarge the people's nerve sensitivity to some events to attract attention. Therefore, the user's sentiment features can reflect current sentiment and attitude to a certain extent. The authors draw a chart for visual analysis to verify the correlation between users' sentiment features and stances. Positive sentiments rarely appear in support and questioning stances (see Figure 1), and negative sentiments account for the highest proportion of sentiment tendencies in the other three stances except for comments. The reason is that once fake news is reported, it will affect the sensitive nerves of the public, and the negative sentiments of netizens will rise. The public will turn to condemn the media as the voices of query and opposition gradually emerge. Therefore, the authors use the sentiment tendency in the comments to represent user sentiment features to assist the judgment of stance.

#### 3.2.2. Cognition Stance

**Figure 2:**
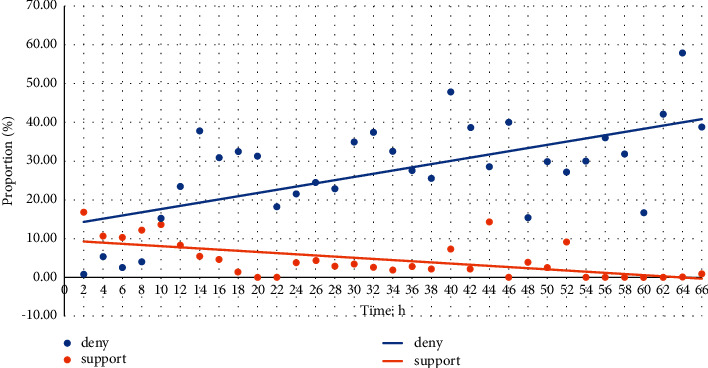
Distributions of comments belonging to support and deny class over time.

After the original microblog is released, more public will participate in the comments over time. When a piece of fake news emerges, people tend to express their views according to the information received at present to support, query, or deny this proposition. However, users receive more comprehensive information and will comment below the source microblog to feedback their views on the proposition over time. For further analysis of this inference, the authors conducted a correlation analysis between cognitive characteristics and stances changing, and the results are shown in Figure 2. After the original fake news release, the proportion of support stances in comments decreased over time, whereas the proportion of deny stances increased. Therefore, time is used to represent the user cognition feature, which is encoded as the difference (in seconds) between the posting time of the target microblog comment and the source microblog.

## 4. Research Method

Given a rumor event, the event contains the source microblog (original rumor microblog) and a series of derivative topics-related microblogs. The format of each microblog comment is *C*={*u*, *t*, *ss*, *cs*}, where *u* is user information, *t* is the text created by users when they publish comments, *ss* is the sentiment tendency, and *cs* is the difference between the posting time of the target microblog comment and the source microblog.

### 4.1. Text Encoder

**Figure 3:**
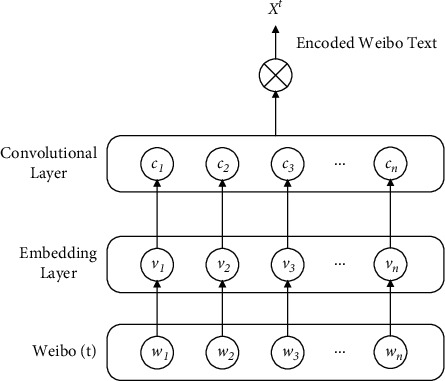
Text encoder.

Each target comment is first encoded by a TextCNN-based [20] text encoder, as shown in Figure 3.

- A comment text *t* is represented as a set of words *t*={*w*_1_, *w*_2_, *w*_3_,…, *w*_*n*_}
- Each word is embedded in a low-dimensional space and represented as a word vector {*v*_1_, *v*_2_, *v*_3_,…, *v*_*n*_}
- The text feature *c*=[*c*_1_, *c*_2_, *c*_3_,…, *c*_*n*_] is obtained by the convolution layer
- Finally, the microblog comment is denoted as *X*^*t*^

### 4.2. Sentiment Signal Encoder

Furthermore, the authors use the sentiment tendency of the comments to represent the sentiment feature *t*_*ss*_. The authors encode user sentiment features as the emotional polarity {positive, neutral, negative}, and the one-hot encoding method is used to obtain the sentiment feature representation *SS*^*t*^.

### 4.3. Cognition Signal Encoder

Furthermore, the user cognition feature *t*_*cs*_ is encoded as the difference (in seconds) between the posting time of the target microblog comment and the source microblog. The authors use the normalized data processing method to obtain cognition feature representation *CS*^*t*^.

### 4.4. Comment Stance Detection Model Based on User Feature Fusion

**Figure 4:**
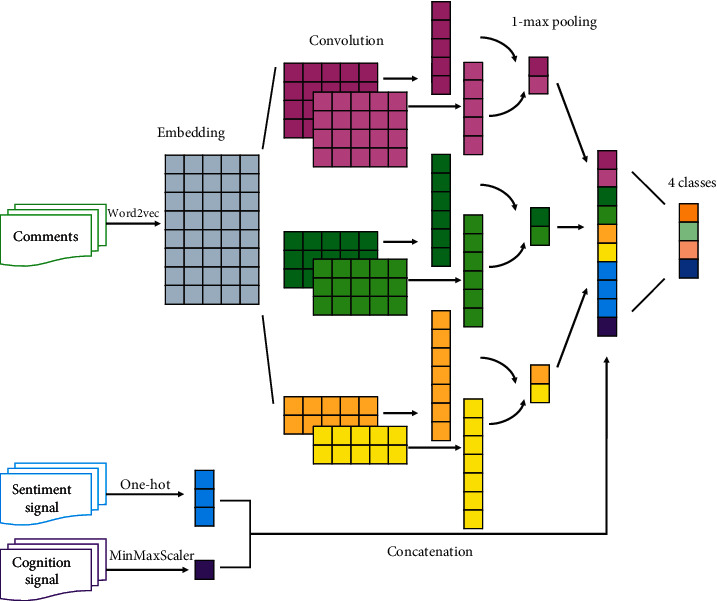
FF-stance (feature fusion-stance): comment stance detection model based on user feature fusion.

Based on the above process description, the authors construct a comment stance detection model FF-Stance (feature fusion-stance) based on user feature fusion (see Figure 4).

**Figure 5:**
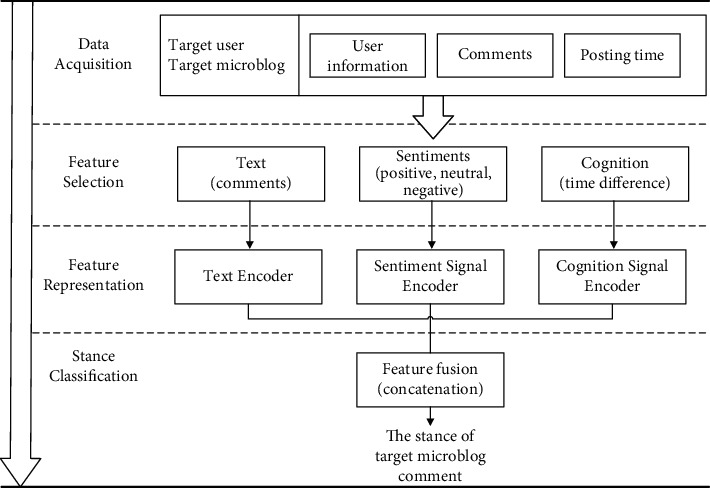
The experimental process.

The experimental process is shown in Figure 5.

- Text representation: the comment is encoded with a TextCNN-based text encoder
- User feature representation: user sentiment feature is encoded as a 1*∗*3 one-dimensional vector using the one-hot method, and user cognition feature is represented using the time difference and normalized
- Feature fusion: the user sentiment feature and cognition feature are concatenated with the text feature vector based on the early fusion method in the pooling layer
- Stance detection: the fused features are sent to the fully connected layer for prediction

## 5. Results and Discussion

### 5.1. Data Acquisition and Preprocessing

By using the hot event “Rumors of illegal soaking antibacterial agent for Wuming fertile orange” as an example, the authors dig in the comments on the relevant microblogs of the media accounts involved in the event (such as The Paper, China Three Agriculture Release, as well as China News Network). After filtering out duplicate and invalid data, the authors collect 9,348 pieces of data in the format of ID, time, and comment text. The stance marking rules are shown in Section 3.1.

Data preprocessing requires the following three steps:

- Chinese text preprocessing: Chinese word segmentation based on statistics has become the mainstream method, with the establishment of large-scale corpus and the continuous development of statistical machine learning methods. Therefore, the authors use jieba for Chinese word segmentation. Because comment text contains many symbols and words unrelated to stance features, such as “@username,” it must be manually processed.
- Manual annotation: sentiment, as an important feature, is added to the stance detection model. The authors divide user sentiment features into {positive, neutral, negative} and stance labels into {support, comment, query, deny}.
- User cognition feature preprocessing: time is an important reference for stance detection. The authors calculate the time difference (in seconds) between the posting time of the target microblog comment and the source microblog to represent the user cognition feature, which changes over time.

The fields and attributes of the processed data are shown in Table 1.

### 5.2. Experimental Method and Parameter Setting

#### 5.2.1. Experimental Methods

- Data set division: the authors use stratified random sampling without replacement to extract 60%, 20%, and 20% examples from the entire dataset, as the training, validation, and test sets, respectively. The detailed statistics of the dataset are shown in Table 2.
- Models: considering the current deep neural network for text classification, the authors choose TextCNN as the stance detection model and compare the performance with other applicable models (including TextRNN [21], TextRCNN [22], and DPCNN [23]).

#### 5.2.2. Experimental Parameter Setting

- Public parameter settings: dropout = 0.5, batch_size = 32, pad_size = 32, learning_rate = 1*e* − 2, require_improvement = 1000, and num_epochs = 15
- Specific parameters of each model are shown in Table 3

### 5.3. Experimental Results and Analysis

#### 5.3.1. Evaluation of FF-Stance

First, the authors choose TextCNN, TextRNN, TextRCNN, and DPCNN as experimental objects to compare the performance of the current deep neural network model for text classification in the stance detection task based on text, as is shown in Table 4. Time complexity (Flops) is the number of operations of the model, which determines the training/prediction time of the model. If the time complexity is too high, it will consume a lot of time on model training and prediction, which makes it difficult to quickly verify the idea and improve the model so as to predict quickly. The space complexity (Params) is the total number of parameters that need to be trained in the network model. To avoid dimensional disaster, the more parameters of the model, the greater the amount of data required to train the model, while the dataset in our experiment is small, which may lead to overfitting of the model. Among the several basic text classification models used in this experiment, RNN does not have an advantage in running speed because it requires more parameters. The time and space complexity of Text CNN are both small, which enables fast model training and prediction in the task of position detection.

Furthermore, the authors introduce user sentiment and cognition features into the stance detection task. The convolution layer is used as the encoder of the comment text, and the processed user features are concatenated with the text feature output using the pooling layer and then input to the full connection layer for prediction. The classification effect of the model is compared based on different combinations of feature structure variants and models, as is shown in Table 5. The results demonstrate that using convolution layers as the text encoder and concatenating user sentiment features helps to capture user sentiment features in the stance detection task and assist in the judgment of user comment stance; using the convolution layers as the text encoder and concatenating temporal features helps to capture user cognition features in the stance detection task, thereby improving the effect of stance detection tasks. The proposed stance detection model based on user feature fusion shows better performance in the user-comment stance detection task along with the optimization of the two abovementioned aspects.

**Figure 6:**
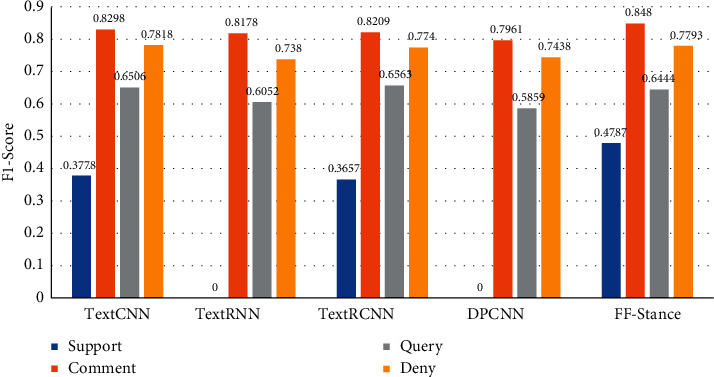
Classification results based on different models in different stances.

The experimental results show that the classification effect based on multidimensional feature fusion has been significantly improved. The classification results of different text classification models in different stances are shown in Figure 6. It shows that after the fusion of user sentiment and cognition features, the classification effect of support stance has been significantly improved. Simultaneously, the classification effect of other stance classes has also been improved to a certain extent. The results demonstrate that the two user features extracted in this study can capture different information of microblog comments, which helps to judge the stance from different dimensions and improve the performance of the stance detection task.

#### 5.3.2. Analysis of Stance Classification Results

**Figure 7:**
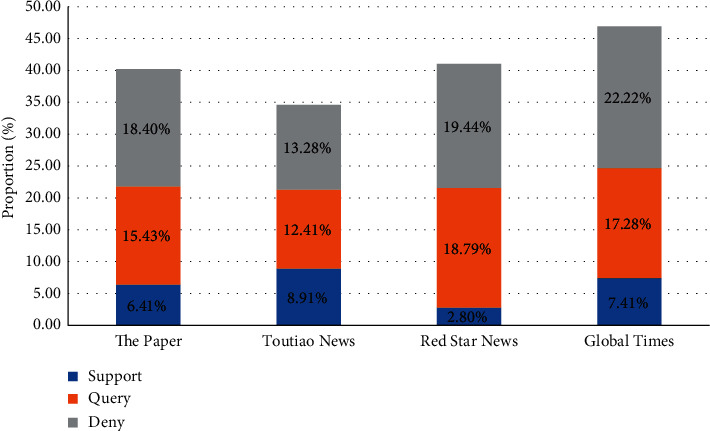
User stance on different media.

To analyze the distribution of netizens' positions in the comments of online media microblogs, the authors take several media that participated in the dissemination of fake news in the incident of “Rumors of illegal soaking antibacterial agent for Wuming fertile orange” as the research object for further discussion, including The Paper, TouTiao News, Red Star News, and Global Times.  Figure 7 shows that few netizens express approval in the comments of microblogs released by the four media involved in fake news dissemination, but most of them show a stance of query or deny. Such dissatisfaction affects the media's credibility, undermines the media's image, and hinders the healthy development of the Internet. In this new media era, where content is critical, grand standing content can attract a short flow. However, because online media is an important part of public opinion dissemination, it should shoulder a significant social responsibility, draw experience from user comments, provide users with better content, enhance user-content perception experience, and create a healthy network communication environment.

## 6. Conclusions

This study extends stance detection from text classification to classification based on multidimensional feature fusion. Considering the influence of user sentiment and cognition on stance, the authors develop a comment stance detection model based on user feature fusion, which fuses user sentiment feature, user cognition feature, and text feature. The performance of the model is compared and evaluated through experiments and theoretical analysis. The experimental results show that the stance detection model based on user feature fusion performs better. However, because the authors are conducting poststudy, behaviors, such as deleting posts, can result in data loss. Therefore, the data collected are unbalanced, and this defect affects the training of the model. Additionally, considering the continuous optimization and diversification of social networking platform functions, the forms of user-generated content are becoming increasingly complex. How to fully incorporate multidimensional features into public opinion mining research is an issue that requires future research.

## Figures and Tables

**Table 1:** Field attributes.

Field	User ID	Comment	Sentiment	Time	Stance
Attribute	Textual	Textual	Nominal	Numeric	Nominal

**Table 2:** The detailed statistics of the dataset.

Dataset	Class	Train size	Dev. size	Test size
Comments	Support	278	93	93
	Comment	3931	977	977
	Query	934	312	312
	Deny	1465	488	488

Total	4	5608	1870	1870

**Table 3:** Specific parameters of various models.

Models	Parameters
TextCNN	filter_sizes = (2, 3, 4), num_filters = 256, pooling = Max
TextRNN	rnn = “lstm”, num_layers = 2, hidden_size = 128, and bidirection = True
TextRCNN	rnn = “lstm”, num_layers = 1, hidden_size = 256, bidirection = True, and pooling = Max
DPCNN	filter_size = 3 and num_filters = 250

**Table 4:** Performance of different text classification models.

Models	Accuracy (%)	Flops (G)	Params (M)
TextCNN	76.20	2.63847936	0.699658
TextRNN	74.39	3.43965696	0.838154
TextRCNN	75.35	4.69865984	1.150914
DPCNN	72.99	3.556864	0.41551

**Table 5:** Performance of various models and architecture variants of FF-stance.

Variation of feature architecture	Models	Accuracy (%)
Text encoder	TextCNN	76.20
	TextRNN	74.39
	TextRCNN	75.35
	DPCNN	72.99

Text encoder + sentiment signal encoder	FF-stance^−^	77.17
Text encoder + cognition signal encoder	FF-stance^−^	76.95
Text encoder + sentiment signal encoder + cognition signal encoder	FF-stance	78.61

## Data Availability

The data used to support the findings of this study are available from the corresponding author upon request.
